# Insights into the Possible Impact of PFAS on Phosphatidylcholine Metabolism and Membranes through Molecular Dynamics Simulations

**DOI:** 10.1021/acsenvironau.5c00299

**Published:** 2026-05-11

**Authors:** Lorenzo Pedroni, Chiara Dall’Asta, Luca Dellafiora

**Affiliations:** † Department of Food and Drug, 9370 University of Parma, Parma 43124, Italy

**Keywords:** PFAS, computational toxicology, mechanisms of action, membrane fluidity, phosphatidylcholine

## Abstract

Poly- and perfluoroalkyl substances (PFAS) are pollutants disrupting lipid metabolism and cell membrane integrity with unclear molecular mechanisms. Molecular docking and molecular dynamics (MD) simulations were used to investigate whether the perfluorinated analogue of palmitic acid (PFHxDA, parametrized through SwissParam) could enter membrane lipid metabolism and get embedded in cell membranes, possibly interfering with their properties. The possible role of PFHxDA as substrate for enzymes involved in the biosynthesis of membrane lipids was studied by investigating its interaction with two enzymes belonging to the phosphatidylcholine biosynthetic pathway, Acyl-CoA synthetase 5 and Choline Phosphotransferase 1, through 200 ns MD simulations. PFHxDA and metabolic derivatives displayed binding and dynamic behaviors comparable to native substrates, suggesting they might be either substrates or inhibitors. Concerning membranes, PFHxDA-containing lipids showed altered bilayer observables such as cholesterol packing, area per lipid, membrane thickness, and membrane density during 300 ns MD simulations, possibly affecting membrane fluidity and organization. Although theoretical, these results provide hypothesis-generating yet compelling mechanistic insights into PFAS-mediated disruption of lipid metabolism and membrane integrity, prioritizing specific experimental follow-up.

## Graphical abstract

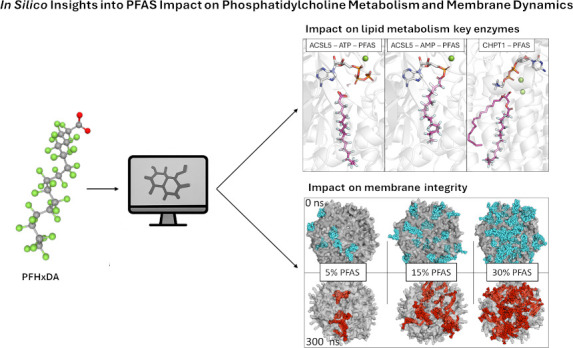

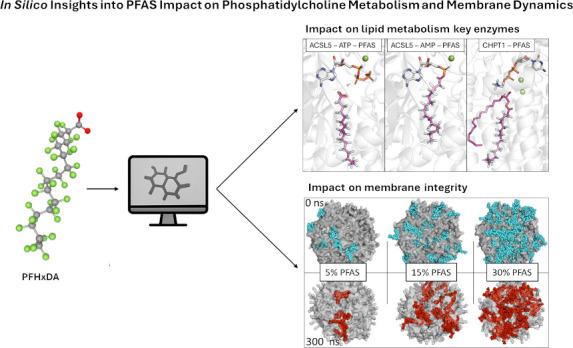

## 1 Introduction

Poly- and perfluoroalkyl substances (PFAS) are environmental pollutants containing at least one fully fluorinated methyl or methylene carbon atom,^1^ prone to biomagnification and bioaccumulation due to their stability,^2–5^ to which humans are chronically exposed.^6^

PFAS exert toxic effects including nephrotoxicity, hepatotoxicity, and disruptive actions on the endocrine system and lipid metabolism.^7,8^ The latter still requires mechanistic clarifications, although increasing evidence points to PFAS-dependent perturbation of lipid homeostasis, possibly inducing liver steatosis.^9^ Recent mechanistic and omics-based studies further corroborated PFAS-induced alterations in triglyceride accumulation, interaction with peroxisome proliferator-activated receptor alpha, a regulator of lipid metabolism,^10,11^ and general disruption of lipid homeostasis.^12–14^ Also, PFAS may alter biological membranes impairing their integrity and functionality.^15,16^ Last, evidence from bacteria highlighted PFAS can be substrates of Acyl-CoA synthetase, a key enzyme in membrane lipids metabolism, though with low reaction yields.^17–19^ These findings provide an evidence-based, mechanistic starting point for further investigation as acyl-CoA synthetases share conserved structural, functional and catalytic features across prokaryotes and eukaryotes.^20–22^ Since mammalian long-chain Acyl-CoA synthetase is crucial for membrane lipid metabolism,^22^ the present work addressed the possible role of PFAS as substrates of human Acyl-CoA synthetase, investigating the mechanistic underpinnings and subsequent potential effects on membrane mechanics.

Acyl-CoA synthetase activates fatty acids through their conjugation to coenzyme A (CoA) enabling their participation in membrane phospholipid anabolism.^23^ Although PFAS reactivity is low, their capacity to serve as Acyl-CoA synthetase substrates has been demonstrated.^17–19^ The prolonged biological residence time of PFAS in living organisms might provide a critical temporal dimension for kinetically unfavorable reactions, especially for humans having elimination half-lives spanning years to decades and negligible metabolic clearance. Chronic exposure, coupled with bioaccumulation and biomagnification, might enable low-efficiency enzymatic processes to achieve appreciable PFAS acyl-conjugates formation, with possible consequences for lipid metabolism and membrane functionalities.

To test this hypothesis, we employed computational tools, increasingly applied as self-standing analytical frameworks in environmental and toxicological sciences,^24,25^ that already succeeded in previous PFAS-related studies.^26–28^ As a target case study, we investigated perfluorohexadecanoic acid (PFHxDA), the perfluorinated counterpart of palmitic acid (PA), and 1-oleoyl-2-palmitoyl-phosphatidylcholine (POPC) as model membrane phospholipid. Although not necessarily representative of the whole PFAS class, it was particularly suitable for testing the proposed hypothesis providing a mechanistic proof of concept. Specifically, the palmitate-like scaffold allowed a methodologically controlled comparison with the native palmitate containing systems. For these reasons, the investigations included: (i) PFHxDA incorporation in POPC, studying the first and last key anabolic reactions (catalyzed by Acyl-CoA synthetase 5 and Choline Phosphotransferase 1, respectively) (Figure 1); (ii) effects of PFHxDA-containing POPCs on membrane through observables such as cholesterol SASA, membrane density, area per lipid, and membrane thickness.

**Figure 1:**
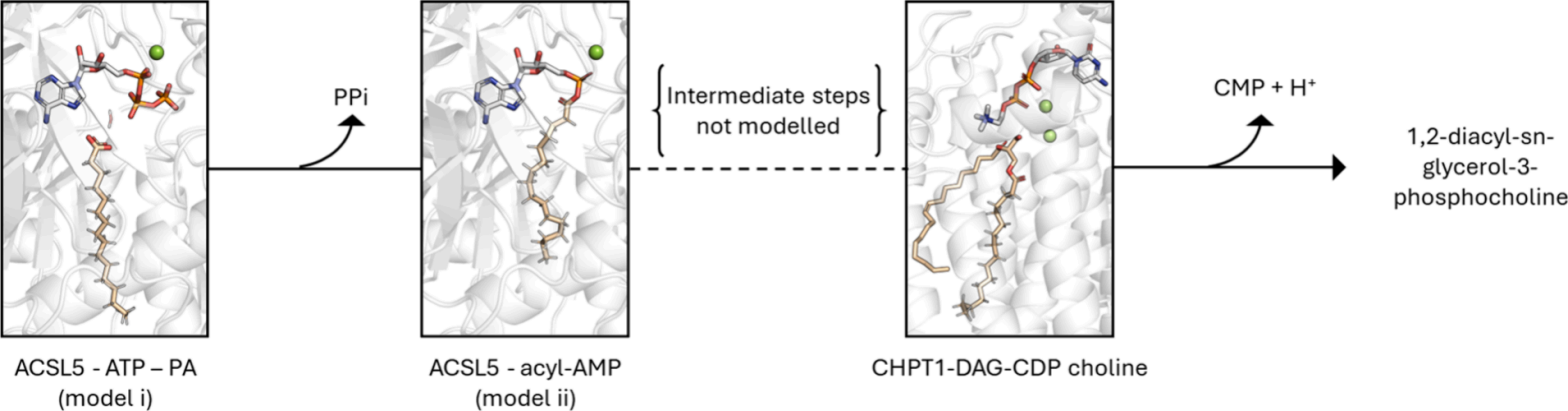
Scheme of the multistep reaction catalyzed by ACSL5, as per ref 29, and CHPT1. The (i) and (ii) are assigned according to the ACSL5 models described in the Materials and Methods and indicate the reaction steps modeled in this study.

## 2 Materials and Methods

### 2.1 Protein Systems Modeling

#### 2.1.1 Acyl-CoA Synthetase 5 (ACSL5)

Based on the multistep reaction catalyzed by ACSL5,^29^ two models were generated to approximate reaction-relevant structural conformations: (i) the precatalytic ternary complex where palmitic acid (PA) is approaching ATP at the ACSL5 catalytic site and (ii) a model of the intermediate binary complex where ACSL5 is bound to PA-AMP intermediate.

Model (i): ACSL5 3D structure was obtained from AlphaFold (Q9ULC5; https://alphafold.ebi.ac.uk/), which showed high model confidence (pLDDT > 90 for the globular region). The conserved water molecule proximal to the ATP phosphate, relevant for substrate recognition processes,^29^ was added by aligning model (i) to the bacterial ACSL structure (PDB ID 1V25) and merging water 1801. The ACSL5 model was used for molecular docking to obtain the PA-ACSL5 architecture of binding. GOLD (Genetic Optimization for Ligand Docking; v. 2024), a software which already succeeded in defining protein-PFAS interactions,^27^ was used. The binding site was set as a 10 Å radius sphere around the retained water molecule, ten different poses were generated, scored via the internal scoring function PLPscore and the best scored one was carried forward to subsequent analysis (the higher the score, the more reliable the binding architecture, as per manufacturer's declaration; https://www.ccdc.cam.ac.uk). At the time of analysis (last PDB access 30th March 2026), suitable experimental reference structures cocrystallized with comparable ligands were not available to validate docking poses either through native-ligand redocking or benchmarking against experimentally resolved complexes. However, the cavity consists of a narrow tunnel in which fatty acids and structurally related congeners have limited conformational freedom,^29^ thereby reducing the likelihood of adopting improper binding geometries. Model (ii) was obtained by aligning the ACSL5 AlphaFold model to the ACSL-myristoyl-AMP complex (PDB ID 1V26). The myristoyl-AMP intermediate was extended by C_2_H_5_ at C14 position to form palmitoyl-AMP via PyMol (v. 2.5.0) and merged.

#### 2.1.2 Choline Phosphotransferase 1 (CHPT1)

Human CHPT1 model (UniProt AC Q8WUD6) was based on the human cryo-EM structure bound to POPC (PDB ID 9UET; chain A). To simulate the POPC formation, the structure was edited via PyMol (v. 2.5.0) converting POPC to its substrate diacylglycerol (DAG) 1-palmitoyl-2-oleoyl-*sn*-glycerol by removing the 2-(trimethylammonio)­ethyl phosphate moiety. Then, cytidine diphosphate-choline (CDP-choline) was added to the generated CHPT1-DAG model by superimposing the *Xenopus laevis* CHPT1-CDP-choline complex (PDB ID 8ERP, overall RMSD 1.1 Å, lowered to 0.6 Å for the residues surrounding CDP-choline; Figure S1, Supporting Information) and merging CDP-choline. RMSD values were calculated through PyMol (v. 2.5.0) with the internal *cealign* function, first using the entire protein structure and then focusing on the residues surrounding (4 Å) CDP-choline.

#### 2.1.3 Complexes with PFHxDA

For all systems, PFHxDA counterparts were generated by replacing PA hydrogens with fluorine atoms through PyMol (v. 2.5.0) . PFHxDA, meant to preserve a comparable carbon chain organization with PA, was parametrized and energy minimized prior to each MD production run (see below for further details).

### 2.2 Molecular Dynamics

Molecular dynamics (MD) simulations were performed using GROMACS (v. 2022.6)^30^ to study the geometric evolution of protein–ligand systems over time. Each ligand-protein complex was parametrized with the CHARMM36 all-atom force field.^31^ While proteins were parametrized using GROMACS internal libraries, the ligands’ CHARMM36 topology and parameters were generated via the SwissParam web-tool (http://swissparam.ch/; force field parameters are reported in the Supporting Information).^32^ Although some limitations cannot be excluded, SwissParam was selected due to its compatibility with the CHARMM36 force field employed for the lipid and protein components, ensuring internal consistency of nonbonded interactions. Its underlying MMFF94-based parametrization has been extensively validated for small organic molecules, including fluorinated compounds, where fluorine atoms are treated within standard Lennard–Jones and partial charge frameworks^33,34^ making it suitable for describing perfluorinated chains in the present systems. This choice is consistent with common practice in major force fields applied to small molecules (e.g., CHARMM, AMBER, and OPLS), where nonbonded interactions are described using Lennard–Jones potentials combined with atom-centered partial charges, which effectively capture the polar character of chemical bonds, including carbon–fluorine bonds (e.g., refs 35 and 36 ). Furthermore, the literature reports independent consensus regarding the applicability of SwissParam to the parametrization of PFAS and recent applications demonstrate reliable performance of SwissParam for perfluorinated compounds in MD simulations.^26,27,37^ All complexes were enclosed in a dodecahedral periodic box, solvated with TIP3P waters and ions (Na^+^ and Cl^–^) at a concentration of 0.15 M. This was followed by energy minimization (steepest descent algorithm, maximum of 5000 steps). Then, isothermal (300 K, 2 ps coupling times) and isobaric (1 bar, 2 ps coupling time) equilibration steps (2.5 ns each) were performed. This was done to obtain equilibrated systems prior to the final 200 ns long MD production step, a time frame consistent with those commonly reported in similar studies.^38–40^

### 2.3 Membrane Modeling

The membrane was built by CHARMM-GUI (www.charmm-gui.org)^41^ and the production via Web server reached completion iteratively setting parameters as follows. The lipid box was set as hexagonal and included a POPC:cholesterol percentage proportion of 70:30 and 90:10 for the upper and lower leaflet, respectively. Of note, given the ongoing debate around cholesterol asymmetric distribution, this choice was not intended to be universally representative but rather physiologically plausible reflecting the well-recognized asymmetry of membrane leaflets in terms of lipid (including cholesterol) composition.^42–45^ Na^+^ and Cl^–^ were added to neutralize the system at 0.15 M, and CHARMM36 and GROMACS were chosen as operating force field and output software compatibility format, respectively. Finally, the system assembly was downloaded in .pdb format, cleaned from water molecules and ions using PyMol (v. 2.5.0), and used as input for local MD simulations. This was done to ensure a uniform preparation pipeline across all simulated systems, both the native POPC:cholesterol membrane and all PFAS-containing systems, as PFAS insertion required manual editing of the coordinate files. Since the coordinate files were manually handled, a complete re-equilibration from scratch was deemed appropriate as an additional precautionary measure and was applied consistently to all systems to ensure methodological uniformity. Importantly, the lipid bilayer coordinates were retained unaltered throughout this process, and thorough re-equilibration (energy minimization, 2.5 ns NVT, 5 ns NPT; see below for further details) was performed prior to the production run to ensure full relaxation of the solvent and ions shell. This two-stage NVT/NPT protocol has been specifically shown to reliably recover equilibrated structural ensembles for lipid membranes even when starting from nonideally equilibrated configurations (e.g., ref 46). The membrane models containing fluorinated C16 POPC analogues (5, 15 and 30%) were generated using a local *ad hoc* script (available as Supporting Information) randomly substituting POPC with fluorinated analogues. It is worth noting that fluorination was performed at the atomic level by replacing hydrogen atoms with fluorine atoms along the acyl chain, with correction of the C–F bond length, while all carbon atom coordinates were retained. This strategy preserves the overall lipid geometry, minimizing perturbations to leaflet packing and lateral lipid organization. Any residual steric strain introduced by the larger van der Waals radius of fluorine relative to hydrogen was relieved during the energy minimization and NVT/NPT equilibration steps prior to the production run (see above). Membrane systems were then processed via GROMACS (version 2022.6) and parametrized with the CHARMM36 all-atom force field.^31^ POPC and cholesterol were parametrized using GROMACS internal libraries, while fluorinated POPC analogues were parametrized using SwissParam web-tool for CHARMM36 all-atom force field (http://swissparam.ch).^32^ The rationale for the choice of SwissParam is detailed above (see Section 2.2) and aligns with previous works simulating PFAS (e.g., ref 37). All membranes were enclosed in a cubic periodic box, solvated with TIP3P waters and an ion concentration (Na^+^ and Cl^–^) of 0.15 M. This was followed by energy minimization to avoid steric clashes (steepest descent algorithm, maximum of 5000 steps). Then, an isothermal (300 K, 0.1 ps coupling time and with a duration of 2.5 ns) and isobaric (1 bar, 1 ps coupling time, applying a semi-isotropic Parrinello–Rahman barostat and with a duration of 5 ns) equilibration steps were performed. This was done to obtain equilibrated systems prior to the final 300 ns long MD production step, in line with simulation lengths commonly applied in studies of this type.^47,48^ In this regard, it should be noted that the present MD simulations are intended to provide a qualitative and comparative picture of the effects of tail fluorination on membrane dynamics, rather than absolute quantitative estimates of thermodynamic or kinetic properties. In this context, the structural observables analyzed (including SASA, membrane thickness, and area per lipid; see below) are well-established metrics that can be reliably observed on nanosecond time scale.^49,50^

Besides SASA, which was computed using the GROMACS internal *sasa* function, standard bilayer observables, namely, area per lipid (APL) and membrane thickness and density, were computed by using GridMAT-MD^51^ over 100 equally time-distributed frames extracted from the last 100 ns of each MD simulation. The used grid was 20 × 20, while the representative atom chosen for the lipid (either POPC or its PFAS derivative) was the phosphorus atom.

## 3 Results and Discussion

### 3.1 ACSL5

ACSL5 is part of the biosynthetic pathway of POPC and catalyzes a multistep reaction to produce acyl-CoA, fundamental for activating fatty acids for transesterification onto glycerol-3-phosphate forming POPC. ACSL5 generates acyl-AMP intermediates prior to final conjugation with CoA. To test whether PFHxDA could behave similarly to PA by forming PFHxDA-CoA, we investigated two models based on available structural information: (i) the precatalytic complex with PA/PFHxDA-ATP and (ii) the PA/PFHxDA-AMP intermediate complexes, both preceding PA/PFHxDA-CoA formation (Figure 2).

**Figure 2:**
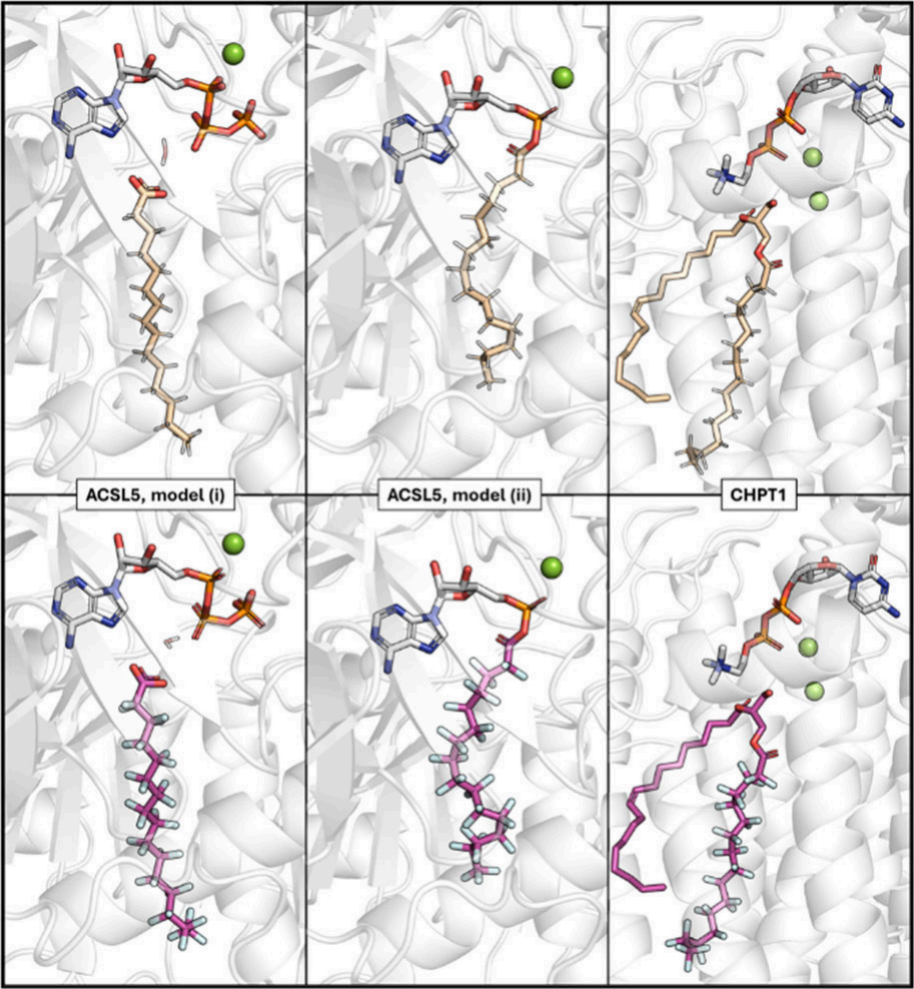
Starting architectures of binding. Proteins are represented in white cartoon, ligands as sticks (beige for fatty acids, magenta for PFAS, and white for cofactors), and ions as spheres. From the left, ACSL5 model (i) i.e. PA/PFHxDA and ATP, ACSL5 model (ii) i.e. PA/PFHxDA-AMP intermediate, and CHPT1 i.e. diacylglycerol-PA/PFHxDA and CDP-choline.

In both models, PA and PFHxDA displayed comparable behavior and stability during the 200 ns MD simulations (Figures S2–S5, Supporting Information). In model (i), ATP’s alpha-phosphate remained similarly close to both PA and PFHxDA throughout the MD simulation. Notably, PFHxDA was found at a shorter distance than PA (0.37 and 0.51 nm, respectively), suggesting that PFHxDA could adopt a spatial arrangement comparable with PA for this specific step. This observation could serve as a first hypothesis supporting possible substrate-like accommodation, though it should be acknowledged that efficient catalysis may rely on additional factors beyond geometrical constraints. In model (ii), PA and PFHxDA displayed similar behavior within the enzyme catalytic site, suggesting a low impact of the more sterically hindered fluorinated chain compared to the native hydrogenated one (Figure S4, Supporting Information). Of note, model (ii) was intrinsically less stable, as expected for a model representing a reaction intermediate (Figure S5, Supporting Information). Nonetheless, this behavior was comparable in both the PA- and PFHxDA-containing complexes.

Mechanistically, PFHxDA and PA behaved comparably, suggesting that PFHxDA could be qualitatively processed by ACSL5 similarly to PA. In line with this observation, a lower, though measurable, capability of ACSL to conjugate CoA to PFAS has been described in previous studies.^18,19^ The capability of PFHxDA and the PFHxDA-containing substrate to persist at the ACSL5 binding site, as described in this study, may also lead to competitive inhibition reducing the availability of ACSL5 for its native substrates, thereby impairing lipid metabolism. However, these interpretations remain hypothesis-generating, as the computational setup applied in this study cannot directly address either catalysis or inhibition. Moreover, chronic lifelong exposure in humans presents a particular scenario that might be worth considering from a mechanistic perspective: the pronounced bioaccumulation of these compounds, particularly marked in adult organisms, could counterbalance their low reactivity and result in PFHxDA-containing membrane lipid precursors at toxicologically relevant concentrations.

### 3.2 CHPT1

CHPT1 catalyzes the final step of *de novo* POPC synthesis transferring the choline phosphate from CDP-choline to the free hydroxyl of a diacylglycerol (DAG), producing POPC and playing a central role in membrane formation, integrity, and functionality. We investigated whether a PFHxDA-containing DAG may act as a CHPT1 substrate (Figure 2). MD simulations showed that the DAG-PFHxDA derivative closely resembled the native DAG substrate. Despite the bulkier fluorinated PFHxDA chain, CHPT1 reached a stable state (Figures S6 and S7, Supporting Information). Moreover, similar to ACSL5, the fluorinated substrate maintained a shorter distance to the CDP-choline cofactor compared with the natural substrate (0.43 and 0.63 nm, respectively). This suggested possible compatibility for PFHxDA derivatives in participating in this reaction.

### 3.3 Membrane

The possible impact of PFHxDA-derivatives incorporation into biological membranes was evaluated by running MD simulations on toy membranes made of POPC, cholesterol and increasing percentages of PFHxDA-containing POPC derivatives—0%, 5%, 15%, 30%. Of note, the chosen fractions are not intended to reflect environmentally realistic concentrations, but they are rather exploratory ranges to test whether there is correlation/coherence between increasing PFAS incorporation and membrane packing/organization. We monitored cholesterol solvent accessible surface area (SASA) variations as a probe of membrane integrity alteration. Increased SASA values denote greater solvent exposure implying reduced cholesterol embedding within the lipid bilayer and lipid-packing capacity, as well as overall enhancement of lipid hydration and water penetration – collectively indicative of structural perturbations altering membrane integrity and functionality.^52,53^ MD simulations displayed a concentration-dependent and progressive increase in cholesterol SASA due to the presence of PFHxDA derivatives (Figure 3). Average SASA values rose from 159.7 Å^2^ at 0% PFHxDA-derivatives to 167.1 Å^2^ at 5%, 170.2 Å^2^ at 15%, and 174.5 Å^2^ at 30% (Table S1, Supporting Information). This trend suggests that cholesterol is less prone to act as a membrane packer in PFHxDA-derivatives-containing membranes supporting a concentration-dependent perturbation and/or reorganization. Such an effect could be further supported by the observed tendency of PFHxDA-derivatives to aggregate (Figure 3), as indicated by the decrease in their average pairwise distances from the beginning to the end of the MD simulations (Table S1, Supporting Information). This observation aligns with the previously described tendency of PFAS to aggregate and form clusters in hydrophilic or hydrophobic environments.^54,55^ To complement cholesterol SASA, additional standard bilayer observables were also inspected. Whole-membrane density profiles (, Supporting Information) displayed a progressive increase in mass density consistent with the PFAS-derivative content, supporting a plausible concentration-dependent bilayer reorganization. Also, membrane thickness showed a modest increasing trend with PFAS-derivative incorporation while average area per lipid remained overall stable up to 15% PFAS-derivative concentration, becoming markedly different at 30% incorporation (Table S2, Supporting Information). The latter should be interpreted as an extreme exploratory condition rather than a plausible real-life scenario. The effects described here also agree with available evidence showing that PFAS may alter membrane properties, including fluidity and permeability,^16,56^ with possible consequences for lipid rafts^57^ and membrane proteins mobility and activity.

**Figure 3:**
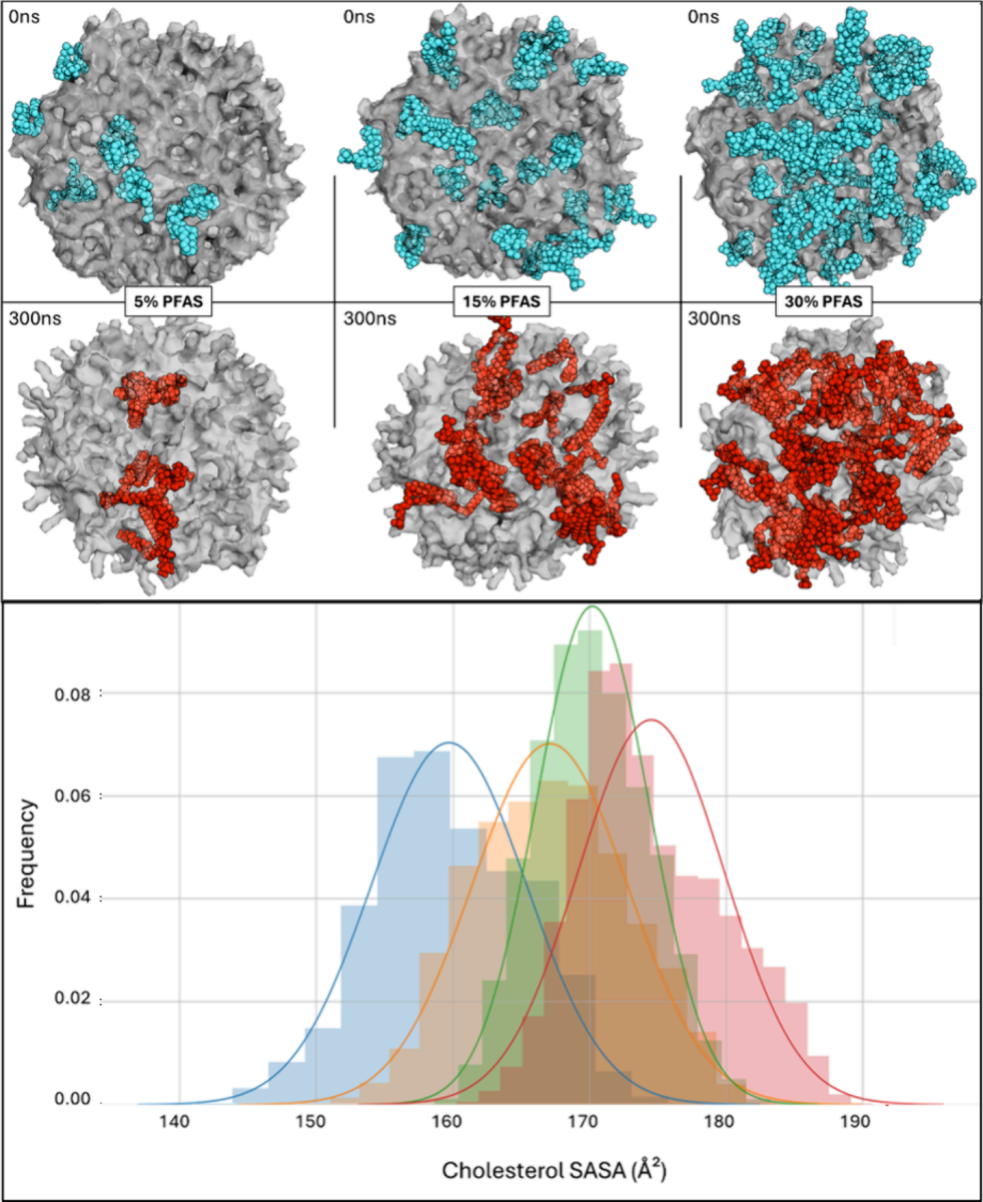
Effect of PFAS-derivatives on membrane integrity and cholesterol solvent exposure. On top, representative MD snapshots of membranes 0 and 300 ns. PFAS-derivatives are shown as spheres (cyan at 0 ns, red at 300 ns) and the membrane is shown as gray surface. On bottom, cholesterol SASA distribution for systems containing 0%, 5%, 15%, and 30% PFAS-derivatives.

Our findings provide a mechanistic, yet exploratory and hypothesis-generating, basis linking lifelong PFAS exposure to impaired lipid metabolism. While PFAS have previously been described as being able to affect cell membranes as individual molecules, we showed that PFHxDA is structurally compatible to participate in key steps for membrane lipid biosynthesis and may enter and perturb cell membranes also as part of complex lipids. Although PFAS display low intrinsic reactivity, their long half-lives and lifelong chronic exposure establish the basis for low-efficiency biochemical processes possibly reaching toxicologically relevant outcomes,^58^ particularly in elderly or dysmetabolic populations. Notably, to our knowledge, PFAS-containing complex lipids have never been described in humans. However, there is no evidence to exclude their existence, and they are likely formed below detection/quantification limits, eluding detection in short-term exposure experimental studies that cannot cope with low reactivity and lifelong exposure. Confirming our mechanistic findings through future dedicated *in vitro*/*in vivo* analyses designed to capture lifelong PFAS bioaccumulation will be essential to clarify how PFAS perturb lipid metabolism under realistic conditions.

## Supplementary Material





## Notes

replace_popc_h_with_f.py, Python script to replace specific hydrogens with fluorine atoms in randomly selected POPC molecules, parameters for ATP (ACSL5 model (i)), parameters for cytidine diphosphate-choline (CHPT1), palmitic acid (ACSL5 model (i)), PA-AMP intermediate (ACSL5 model (ii)), PA_CHPT1.itp, diacylglycerol-PA (CHPT1), PFHxDA-AMP intermediate (ACSL5 model (ii)), diacylglycerol-PFHxDA (CHPT1), PFHxDA (ACSL5 model (i)), and POPC PFAS-derivative (ZIP)
Superimposition of CHPT1 binding sites, additional figures reporting MD simulation results, and observables for membranes (PDF)

## Abbreviations

ACSL5: Acyl-CoA synthetase 5
APL: area per lipid
ATP: adenosine triphosphate
AMP: adenosine monophosphate
CDP-choline: cytidine diphosphate-choline
CHPT1: Choline Phosphotransferase 1
CoA: coenzyme A
DAG: diacylglycerol
GOLD: Genetic Optimization for Ligand Docking
MD: molecular dynamics
OECD: Organisation for Economic Co-operation and Development
PA: palmitic acid
PFAS: poly-/per-fluoroalkyl substances
PFHxDA: perfluorohexadecanoic acid
POPC: 1-oleoyl-2-palmitoyl-phosphatidylcholine
SASA: solvent accessible surface area
