# Supplementary material for: Insights into the Possible Impact of PFAS on Phosphatidylcholine Metabolism and Membranes through Molecular Dynamics Simulations
Source: ACS Environ Au. 2026 May 11;6(4):566–72. doi: 10.1021/acsenvironau.5c00299 (PMC13377507; doi:10.1021/acsenvironau.5c00299)
Supplement: Supplementary file 2 [file vg5c00299_si_002.pdf]

Supplementary Materials

Insights into the possible impact of PFAS on phosphatidylcholine metabolism and membranes through molecular dynamics simulations

Lorenzo Pedroni [1], Chiara Dall'Asta [1], Luca Dellafiora [1,*]

[1] Department of Food and Drug, University of Parma, Parma, 43124, Italy

* Correspondence to: Luca Dellafiora, Department of Food and Drug, University of Parma, Parco Area delle Scienze 27/A, 43124 Parma, Italy. Phone: +39 0521 902073. Email: luca.dellafiora@unipr.it

List of supporting files

- *replace_popc_h_with_f.py*: python script to replace specific hydrogens with fluorine atoms in randomly selected POPC molecules
- *ATP.itp*: parameters for ATP (ACSL5 model (i))
- *CPD.itp:* parameters for cytidine diphosphate-choline (CHPT1)
- *PA.itp:* parameters for palmitic acid (ACSL5 model (i))
- *PA_AMP.itp:* PA-AMP intermediate (ACSL5 model (ii))
- *PA_CHPT1.itp:* diacylglycerol-PA (CHPT1)
- *PFAS_AMP.itp:* PFHxDA-AMP intermediate (ACSL5 model (ii))
- *PFAS_CHPT1.itp:* diacylglycerol-PFHxDA (CHPT1)
- *PFHxDA.itp:* parameters for PFHxDA (ACSL5 model (i))
- *POPC_F.itp*: parameters for POPC PFAS-derivative

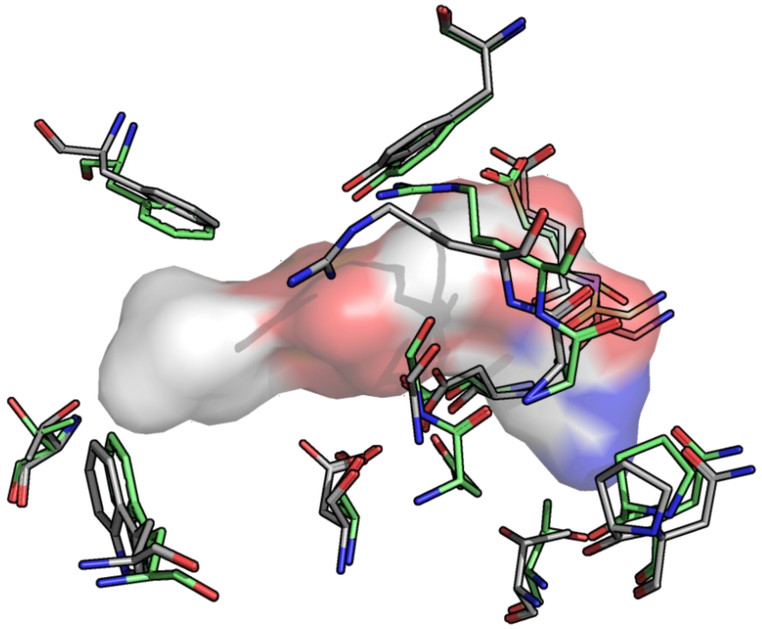

Figure S1. Representation of the superimposition between the residues surrounding (4Å) CDP-choline extracted from PDB ID 8ERP (gray sticks) while residues belonging to PDB ID 9UET (green sticks), used as our model for human CHPT1. CDP-choline is represented in surface.

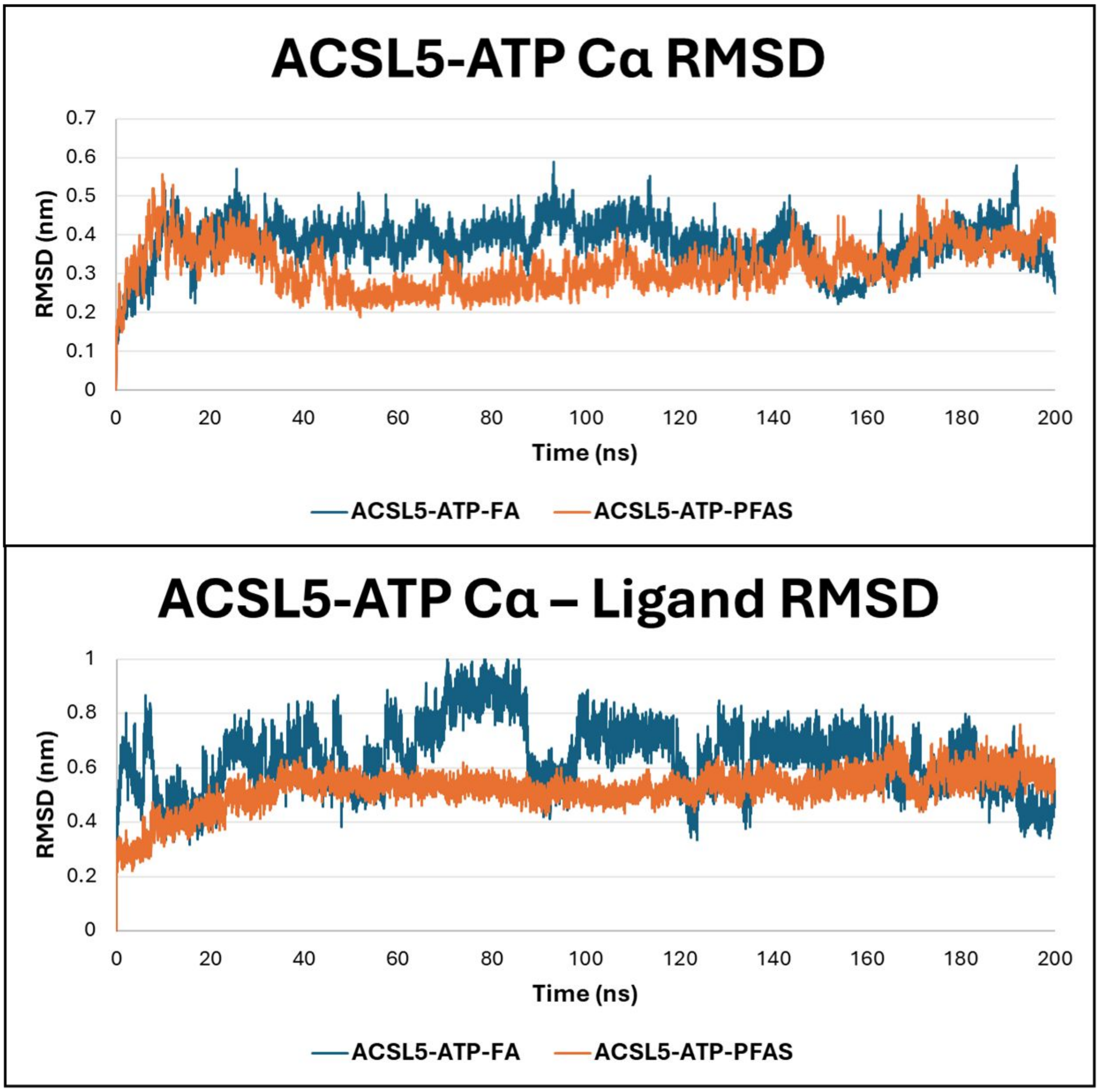

Figure S2. MD results showing ACSL5 RMSD when in complex with ATP and native fatty acid (FA) or PFAS. On the top, ACSL5 Cα RMSD. On the bottom, ACSL5 Cα – FA/PFAS RMSD. Both showed comparable trends.

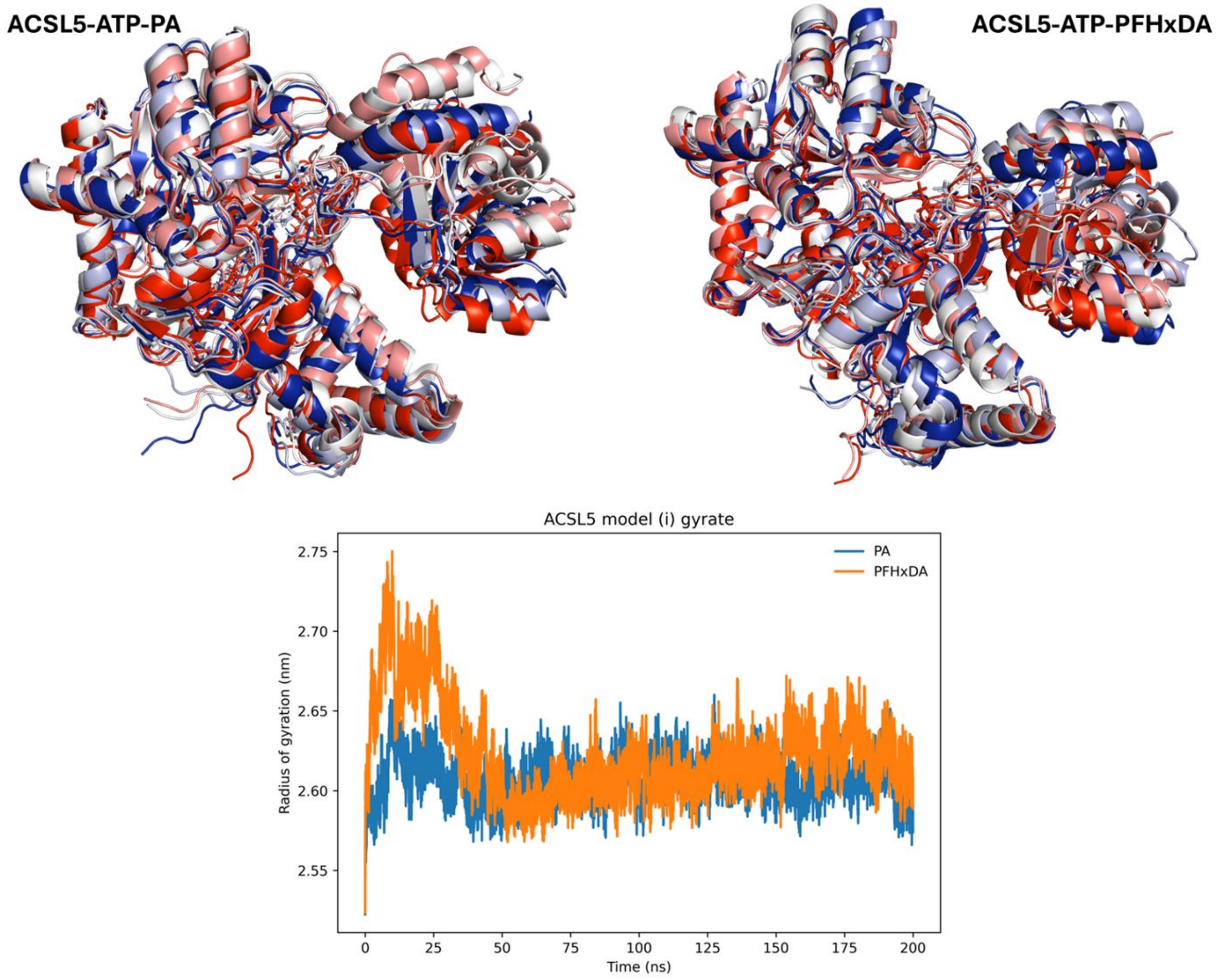

Figure S3. On top, structural superposition of five equally time-distributed frames from 0 to 200 ns for ACSL5-ATP-PA and ACSL5-ATP-PFHxDA. Protein is represented as cartoon and coloured according to the simulation time from red (0 ns) to white (100 ns) to blue (200 ns). The highlighted region indicates the protein domain undergoing visible conformational variability toward the end of the simulation. On bottom, radius of gyration over 200 ns MD simulations computed over protein Cα. Both systems remain globally stable.

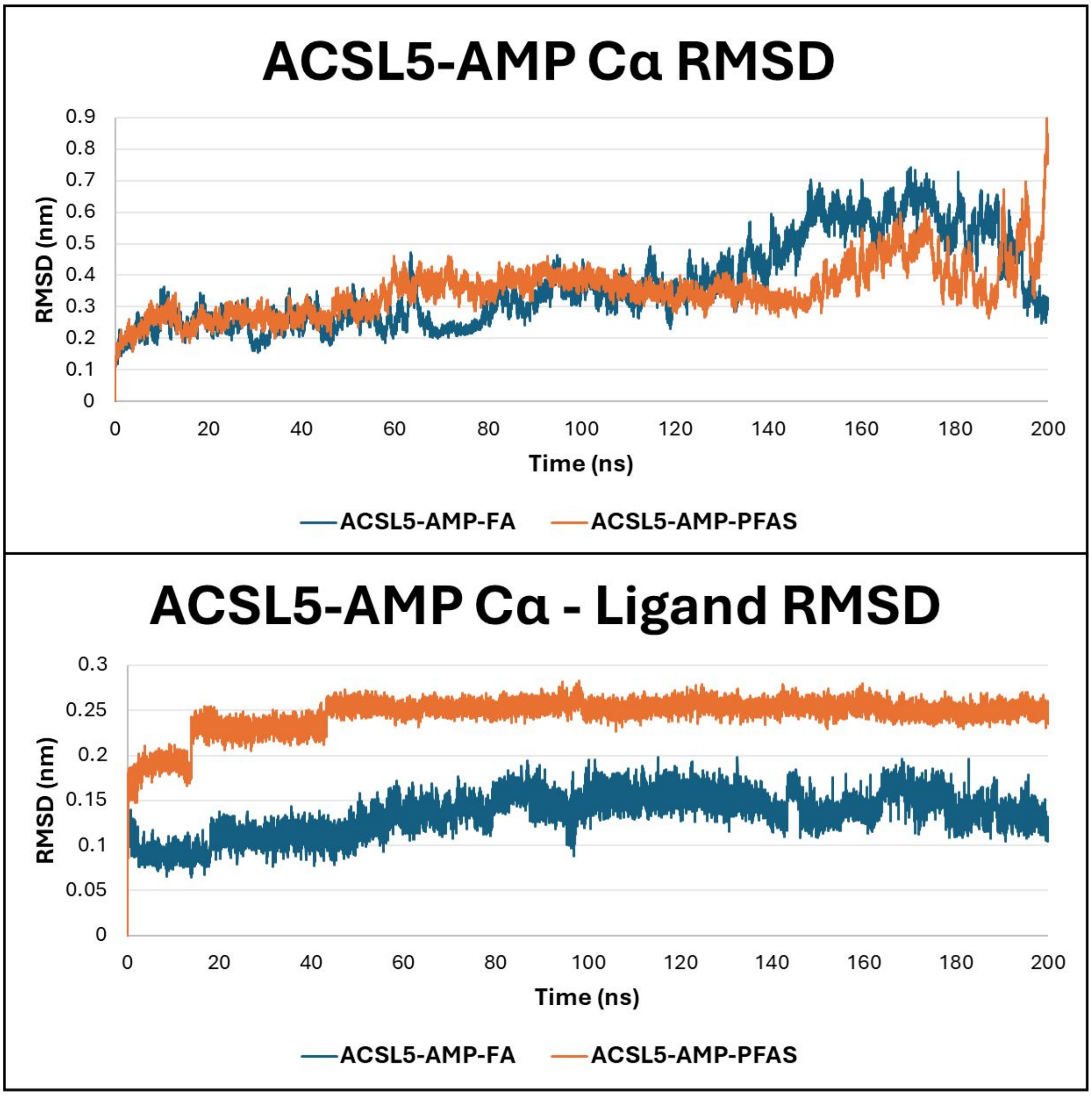

Figure S4. MD results showing ACSL5 RMSD when in complex with AMP complexed with the native fatty acid (FA) or PFAS. On the top, ACSL5 Cα RMSD. On the bottom, ACSL5 Cα – AMP-FA/PFAS RMSD. Both showed comparable trends.

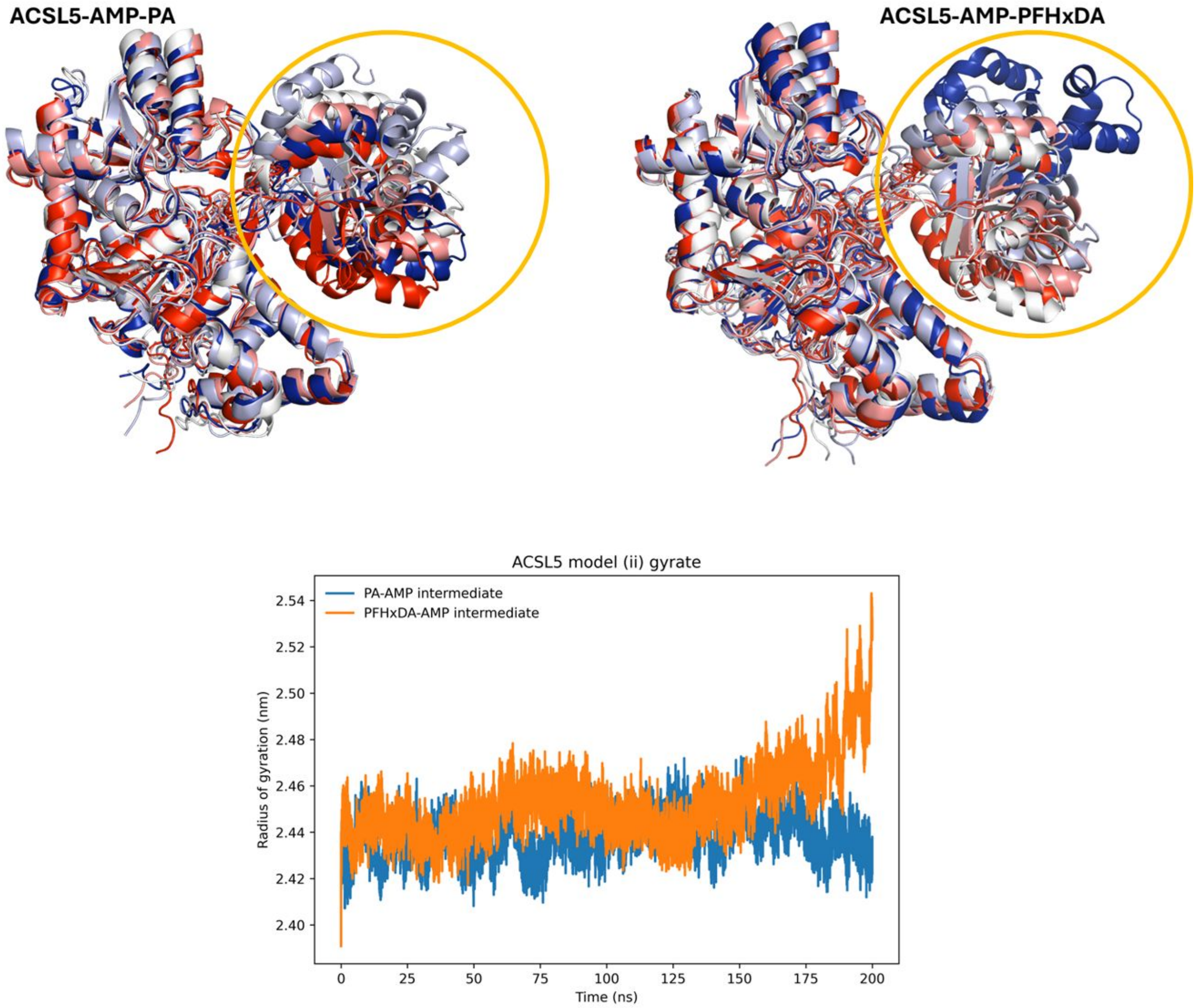

Figure S5. On top, structural superposition of five equally time-distributed frames from 0 to 200 ns for ACSL5-AMP-PA and ACSL5-AMP-PFHxDA. Protein is represented as cartoon and coloured according to the simulation time from red (0 ns) to white (100 ns) to blue (200 ns). The highlighted region indicates the protein domain undergoing visible conformational variability toward the end of the simulation. On bottom, radius of gyration over 200 ns MD simulations computed over protein Cα. Even if both systems remain globally stable, there is a modest rearrangement toward the end of the MD simulation, especially affecting the system with PFHxDA-AMP intermediate.

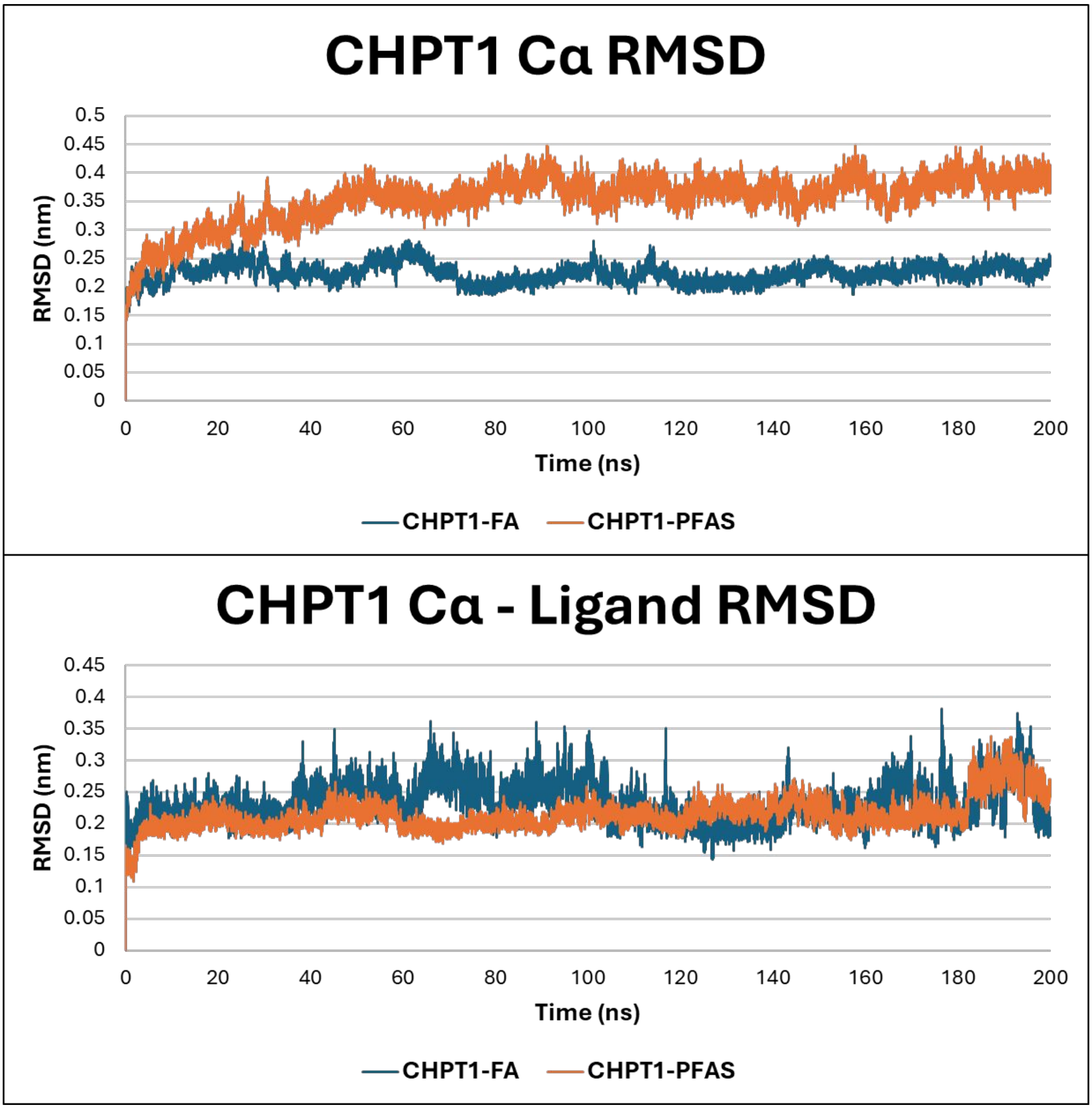

Figure S6. MD results showing CHPT1 RMSD when in complex with choline-fatty acid (FA) or choline-PFAS derivative (PFAS). On the top, CHPT1 Cα RMSD. On the bottom, CHPT1 Cα – FA/PFAS RMSD. Both showed comparable trends.

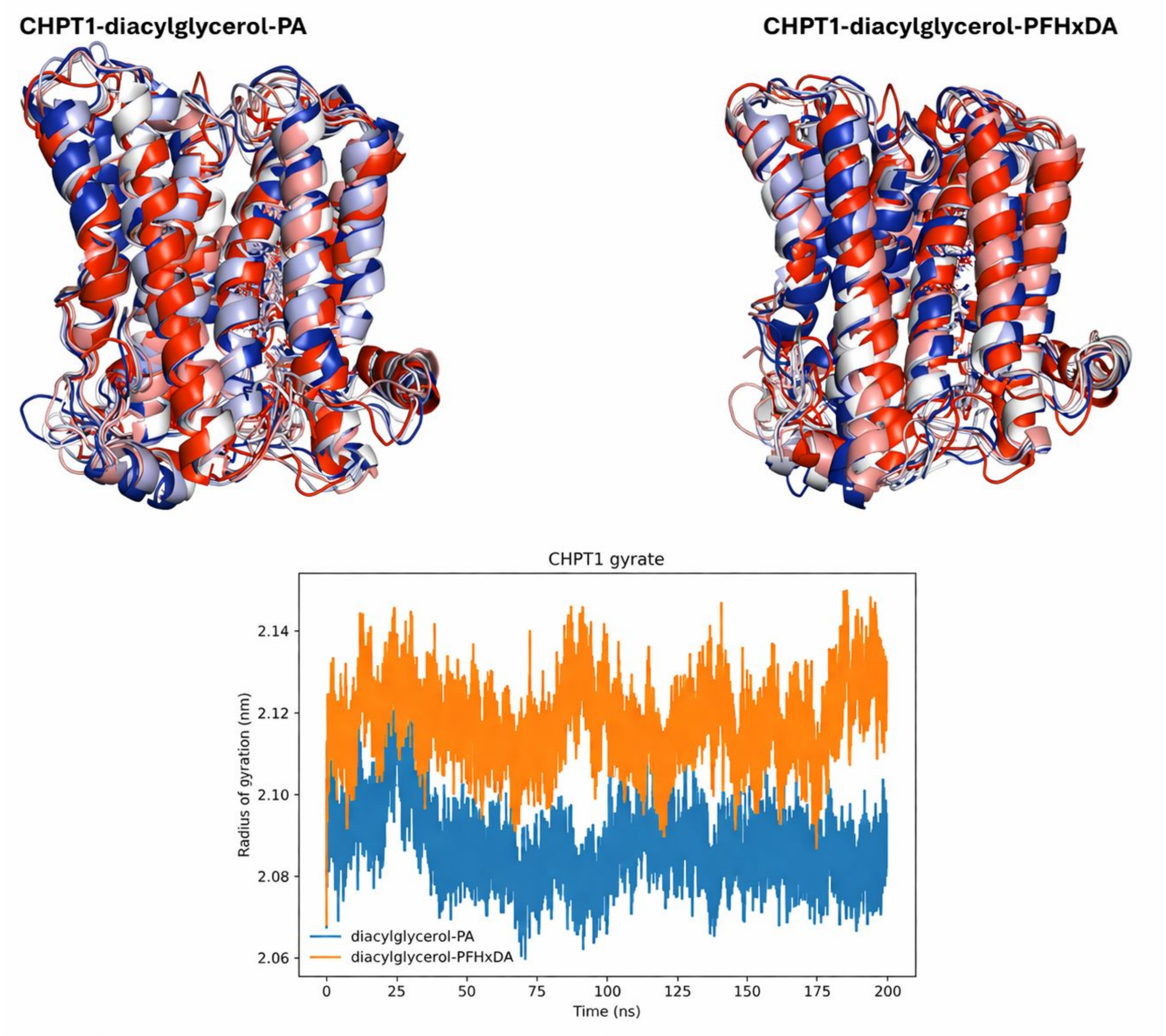

Figure S7. On top, structural superposition of five equally time-distributed frames from 0 to 200 ns for CHPT1-diacylglycerol-PA and CHPT1-diacylglycerol-PFHxDA. Protein is represented as cartoon and coloured according to the simulation time from red (0 ns) to white (100 ns) to blue (200 ns). The highlighted region indicates the protein domain undergoing visible conformational variability toward the end of the simulation. On bottom, radius of gyration over 200 ns MD simulations computed over protein Cα. Both systems remain globally stable.

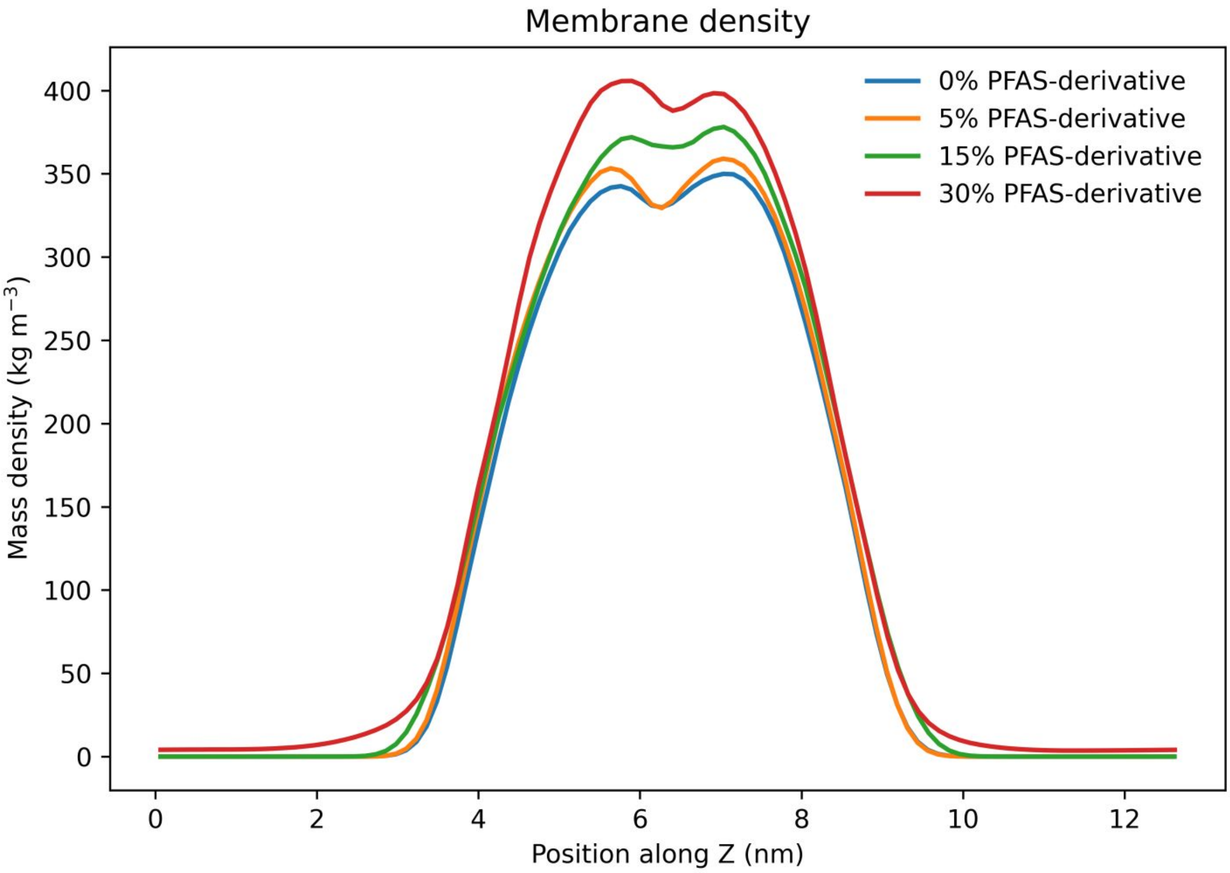

Figure S8. Membrane density profiles along the bilayer normal (Z-axis) for membranes containing increasing percentages, from 0% to 30%, of PFAS-derivatives.

Tables

Table S1. Effect of increasing PFAS-derivative content on cholesterol solvent-accessible surface area (SASA) and PFAS-derivative pairwise distance variation over 300 ns MD simulations.

	**0% PFAS-derivatives**	**5% PFAS-derivatives**	**15% PFAS-derivatives**	**30% PFAS-derivatives**
**Cholesterol SASA (Å²)**	159.7	167.1	170.2	174.5
**PFAS-derivatives pairwise distance variation (Å)**	n.p.	-4.26	-9.66	-4.73

Note: n.p. stands for not performed.

Table S2. Average area per lipid (APL) and membrane thickness for membranes containing increasing percentages of PFAS-derivative phospholipids. Values were computed over 100 equally time-distributed frames extracted from the last 100 ns of the 300 ns MD simulations and are reported as mean ± SD.

	**0% PFAS-derivatives**	**5% PFAS-derivatives**	**15% PFAS-derivatives**	**30% PFAS-derivatives**
**Average APL (Å²)**	160.7520 ± 0.3125	160.8281 ± 0.4279	161.1235 ± 0.3637	272.1925 ± 81.1200*
**Average thickness (nm)**	2.2946 ± 0.0938	2.3533 ± 0.1097	2.3833 ± 0.1149	2.9605 ± 0.6962

Note: * It should be treated as an extreme exploratory condition not representative for a real-life scenario. Of note, this pronounced variability for APL at 30% PFAS-derivative concentration suggests an increased structural variability compared with the lower ones.
